# Cell specific variation in viability in suspension in in vitro Poiseuille flow conditions

**DOI:** 10.1038/s41598-021-91865-1

**Published:** 2021-07-07

**Authors:** Sinead Connolly, David Newport, Kieran McGourty

**Affiliations:** 1 School of Engineering, Bernal Institute, University of Limerick, Limerick, V94 T9PX Ireland; 2 Health Research Institute, University of Limerick, Limerick, V94 T9PX Ireland; 3 School of Natural Sciences, Bernal Institute, University of Limerick, Limerick, V94 T9PX Ireland

**Keywords:** Cellular motility, Cell death, Biomedical engineering

## Abstract

The influence of Poiseuille flow on cell viability has applications in the areas of cancer metastasis, lab-on-a-chip devices and flow cytometry. Indeed, retaining cell viability is important in the emerging field of adoptive cell therapy, as cells need to be returned to patients’ bodies, while the viability of other cells, which are perhaps less accustomed to suspension in a fluidic environment, is important to retain in flow cytometers and other such devices. Despite this, it is unclear how Poiseuille flow affects cell viability. Following on from previous studies which investigated the viability and inertial positions of circulating breast cancer cells in identical flow conditions, this study investigated the influence that varying flow rate, and the corresponding Reynolds number has on the viability of a range of different circulating cells in laminar pipe flow including primary T-cells, primary fibroblasts and neuroblastoma cells. It was found that Reynolds numbers as high as 9.13 had no effect on T-cells while the viabilities of neuroblastoma cells and intestinal fibroblasts were significantly reduced in comparison. This indicates that in vitro flow devices need to be tailored to cell-specific flow regimes.

## Introduction

Suspended cells are commonly used in ex vivo microfluidic techniques such as CAR T-cell therapy, lab-on-a-chip devices and flow cytometry utilizing microchannels of 5–300 $\upmu$m to analyse cells for research or diagnostic purposes, to separate suspended cells^1–5^ or for therapeutic applications^6^. However, little is known about how the fluid mechanics in these channels influence the viability of cells, and few studies have delved into cellular reactions to hydrodynamic forces in microfluidic devices^7,8^, despite the fact that their survival is paramount for the success of patient treatment.

Fluid in a pipe imposes a number of forces and stresses on any particles or cells suspended in it. Poiseuille flow is often characterized by the Reynolds number (*Re*) which can be defined as

1 $$\begin{aligned} Re = \frac{\rho {\bar{U}}{2R}}{\mu } \end{aligned}$$

where $\rho$ is the density of the fluid, ${\bar{U}}$ is the mean fluid velocity, *R* is the radius of the circular channel and $\mu$ is the fluid viscosity. According to Poiseuille flow, fluid shear stress $\tau (r)$ is highest at the channel wall (otherwise known as wall shear stress ($\tau _w$)) where it is defined as

2 $$\begin{aligned} \tau _w=\frac{4{\bar{U}}\mu }{R} \end{aligned}$$

$\tau _w$ decreases towards the channel centre in a circular pipe carrying laminar flow^9^, however, as $\tau _w$ in pipe flow has previously been found to be a poor predictor of cell viability^10^, others, such as the shear stress gradients acting on the surface of the particle ($\nabla \tau _p$), or the shear stress gradient across the channel width also need to be considered^11^. Different methods have been used to examine the effects of fluidic forces on cells in suspension, including cone and plate experiments^12–14^, continuous flow circuits^13,15–21^ and syringe and needle set-ups^13,22–25^. Cone and plate experiments can be used to apply a constant known $\tau _w$ to cells adhered to a plate and while useful for studying the effects of shear on endothelial cells, they do not replicate the $\tau (r)$ that cells suspended in a microchannel would be exposed to, as the shear gradient is constant. Continuous flow circuits, comprising a peristaltic pump and microchannel in an unbroken loop provide a more accurate estimation of cell reactions due to pipe flow over an extended period of time, however it fails to satisfactorily replicate in vivo fluid dynamics^13^. Additionally, it has been shown that peristaltic pumps have a negative effect on cell viability, thereby compromising results^10^. Finally, the syringe and needle set-up provides a good replication of Poiseuille flow, however in the majority of studies, the method is time restricted due to the volume capabilities of both the syringe and tubing.

**Figure 1:**
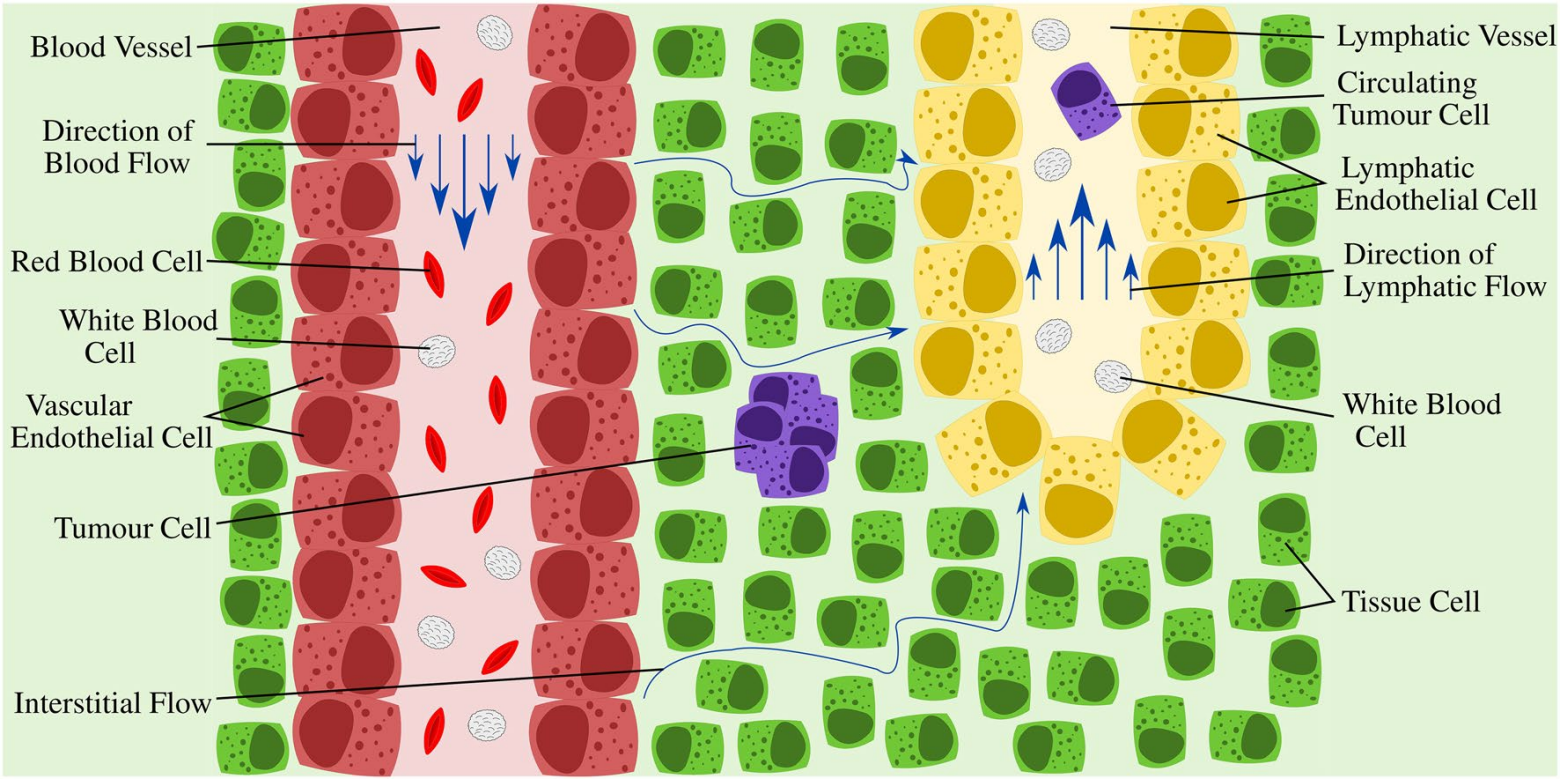
Different flow regimes in the body. High-velocity fluid exits the blood vessels, surrounding the normally static tissue cells, and progressing slowly in the direction of the lymphatic vessels. This is known as interstitial flow. Excess fluid is collected by the lymphatic vessels and returned to the blood. Fluid flow direction is indicated by the blue arrows. Figure was created using Inkscape^31^.

Previous studies carried out by this group, using an adapted syringe and needle set-up over a 24 h period, have found that circulating tumour cells in microchannels occupy inertial positions at the centre of the vessel that shield them from high $\tau _w$ values at the wall^10^, however, as two transformed breast cancer cell lines were the subject of these experiments, it remains questionable whether these findings are broadly applicable to other cell types. Breast cancer cells normally reside in static tissue, however, metastatic varieties possess the ability to invade the local blood or lymphatic vessels, becoming circulating tumour cells^26,27^, as shown in Fig. 1, thereby necessitating a base level of resistance to flow. The findings of this previous study corroborate this hypothesis, where negligible cell death was observed in high-shear flow conditions. While extensive studies have been carried out on the effects of hydrodynamic forces on circulating tumour cells^28–30^, very few to date have examined the same phenomenon in other cells which, unlike metastatic cancer cells may inhabit exclusive environments. These can include suspension in high-velocity fluid flow, such as WBCs and red blood cells (see Fig. 1) or fixed in static tissue, exposed only to very low-velocity interstitial flows such as the tissue cells displayed in Fig. 1.

It has been found, using a syringe and needle set-up, that the viability of primary cells normally suspended in high-velocity fluid flow such as WBCs are unaffected in a 410 $\upmu$m inner diameter tube at flow rates of up to 8 mL/min, however, as previously mentioned, cells were only exposed to these conditions for a matter of milliseconds^7,32^. It would, therefore, be interesting to discern if they, similar to breast cancer cells, possess sustained viability over an extended period of time in high-velocity fluid flows. Furthermore, due to the requirements of adoptive cell therapy and other in vitro assays, there is a need for a comprehensive test that will expose WBCs to microchannel hydrodynamic forces for prolonged durations to determine the limits of their ability to survive^33^.

Additionally, as ordinarily adherent tissue cells can sometimes be analysed in suspension in flow cytometry or lab-on-a-chip assays^34^, it was also considered to be important to analyse the viability of these cells under suspension in microchannel flow conditions, particularly as, to these authors’ knowledge, no previous studies have examined this. Furthermore, new stem cell therapies are continually becoming more widely available, including mesenchymal stromal cell therapies which can be used in the treatment of Crohn’s disease or acute graft versus host disease. While not exposed to such environments in the body, in vitro research and diagnostic processes may compromise their viability, further highlighting the requirement for such an assay. This group has previously successfully utilized such a set-up in order to analyse similar reactions of circulating breast cancer cells^10^, and so this method was again used to question whether these previous observations hold true for primary cells, and other cancer cell types in microchannel applications.

This study investigated the influence that varying flow rate, and the corresponding *Re* has on the viability of circulating cells in laminar pipe flow. Different cells, each hailing from different native fluidic environments, were chosen for this study to examine if there was a difference in their viabilities following exposure to Poiseuille flow. These previously included two breast cancer cell lines, one a benign cell line (MCF-7 cells), ordinarily resident in static tissue, and the other, a metastatic cell line (MDA-MB-231 cells), ordinarily resident in static tissue but with the ability to invade the local vasculature^10^ (see Fig. 1). Investigated in this study were primary T-cells, which would normally be continuously suspended in fluid flow in both the cardiovascular and lymphatic systems, and primary intestinal fibroblasts, whose native environment is in static tissue, exposed only to interstitial flow. Neuroblastoma cells, a cancer cell line, less accustomed to flow in suspension than the previously tested breast cancer cells, with a lower metastatic potential, were also investigated to examine if the high viability rate observed in breast cancer cells is common to other cancer cell types.

## Results

### Viability of T-cells in Poiseuille flow

**Figure 2:**
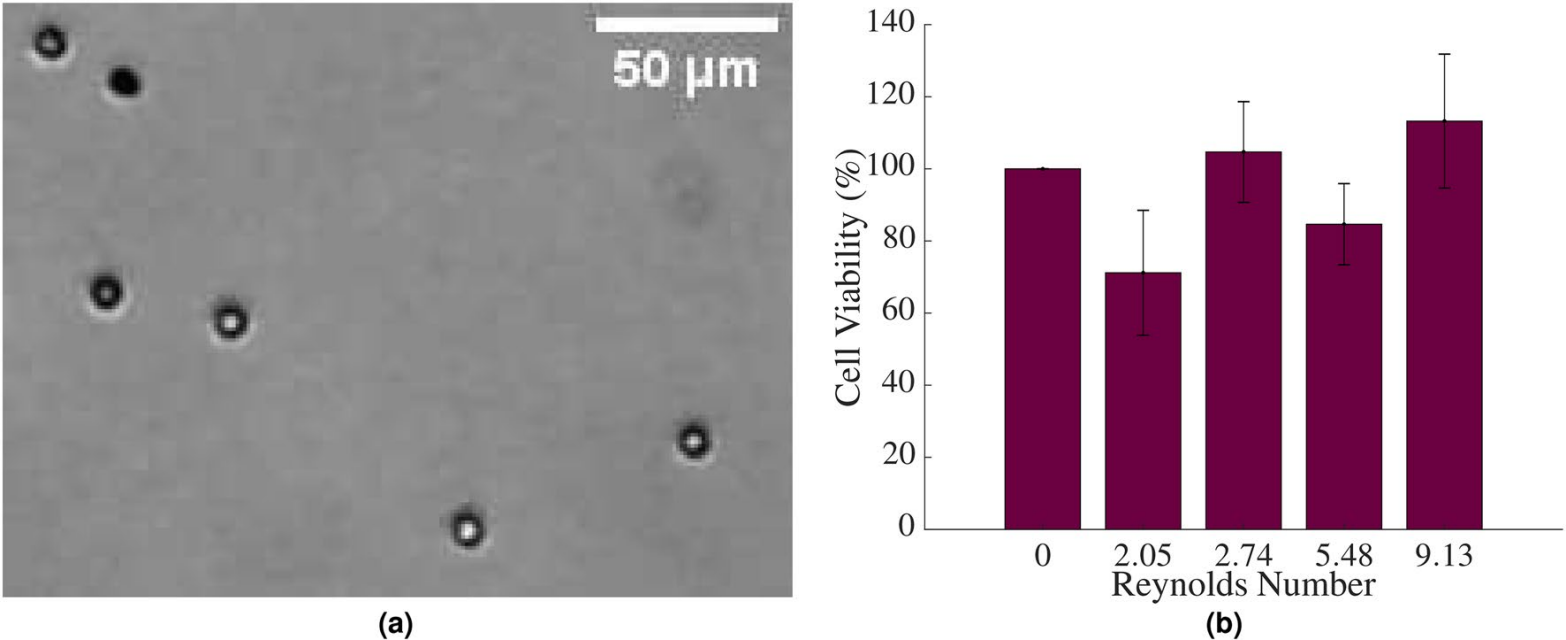
**(a)** T-cells following suspension in Poiseuille flows of $Re=$ 5.48 for 24 h, stained with trypan blue. **(b)** The viability of T-cells in circulation, assessed using MTT. Statistical analysis was conducted using two-way ANOVA. Results were normalised to static cells in suspension (or *Re* of 0). $n=$ 9 replicates, vertical bars represent the s.e.m.

This group previously observed high viability levels in breast cancer cells in Poiseuille flow^10^, and so, it was desired to address whether this was an artefact of transformed cells or was true of all cells under these conditions. To this end, various cell types from different flow regimes were investigated. First, the viability of T-cells, a professional advecting cell (see Fig. 1), exposed to Poiseuille flow for 24 h, was assessed to evaluate if similarly high levels would be observed. Cell viability was assessed using MTT as outlined in ‘Methods’. An image of the T-cells following exposure to $Re=$ 5.48 and stained by trypan blue is shown in Fig. 2a and the results are shown in the bar graph in Fig. 2b.

There was no significant difference between the control viability or the viability of T-cells at any *Re*, according to the MTT assay. The viability of the T-cells was unaltered by an increasing flow rate and therefore increasing *Re*, however there appeared to be a slight decrease in the viability at lower *Re*. At *Re*, typical of the lymphatic system, at 2.74, the viability of the T-cells was 105 ± 14%, while at higher *Re*, more representative of the cardiovascular system, of 9.13, cell viability again remained at 113 ± 19%. These findings are in agreement with physiological levels encountered quite regularly in the cardiovascular system. Additionally, it is interesting to note that their viability is unchanged in laminar flow conditions, typically encountered within the lymphatic system^35^, and the results are consistent with this.

These findings agree with a previous similar study on the viability of leukocytes in a syringe and needle set-up. Following a syringe and needle experiment using a flow rate of 0.3–8 mL/min in a 0.41 mm diameter needle, producing $Re \approx$ 15–413, Suwannaphan et al. found that leukocytes did not experience any change in viability following experiments lasting between 0.0125 and 0.25 ms^7^. This study indicated that a longer experimental time frame, albeit at lower *Re* (2.05–9.13), also had no effect on the viability of the cells. Furthermore, it indicated that a decrease in the channel size of more than 70% did not impact the viability of T-cells in any way, demonstrating their durability in microchannels. This confirms that cell processing protocols that use microchannels to separate and analyse T-cells, have no consequences for the cells’ viability. Additionally, in the preceding study carried out by this group, similar results were found in breast cancer cells; that an increase in flow rate and, consequently, *Re* and $\tau _w$, has very little influence on the cells’ viability^10^.

### Viability of intestinal fibroblasts in Poiseuille flow

All three cell types tested so far possessed unaffected viability levels following exposure in Poiseuille flow. Therefore, it was next evaluated whether primary cells isolated from a flow restricted physiological environment also survived. To this end, the viability of neonatal porcine primary intestinal fibroblasts exposed to Poiseuille flow for 24 h was investigated. Intestinal fibroblasts were chosen as, because they reside in static tissue of the intestinal submucosal space, their native environment is not in suspension at all (see ‘Tissue Cells’ in Fig. 1); and so, is in direct contrast to the native environment of the T-cells. They may, however, be exposed to interstitial flow which can result in $\tau _w$ values of $\approx$ 0.4 Pa^36^, much lower than those investigated in this study. As a reduction in cell anchoring, caused by a decrease in integrin functioning, is known to cause a decrease in cell viability in normally adherent cells^37^, it is necessary to discern if exposure of normally adherent, primary cells to fluid flow would result in different viability to those of cancer cells or natively advecting T-cells.

**Figure 3:**
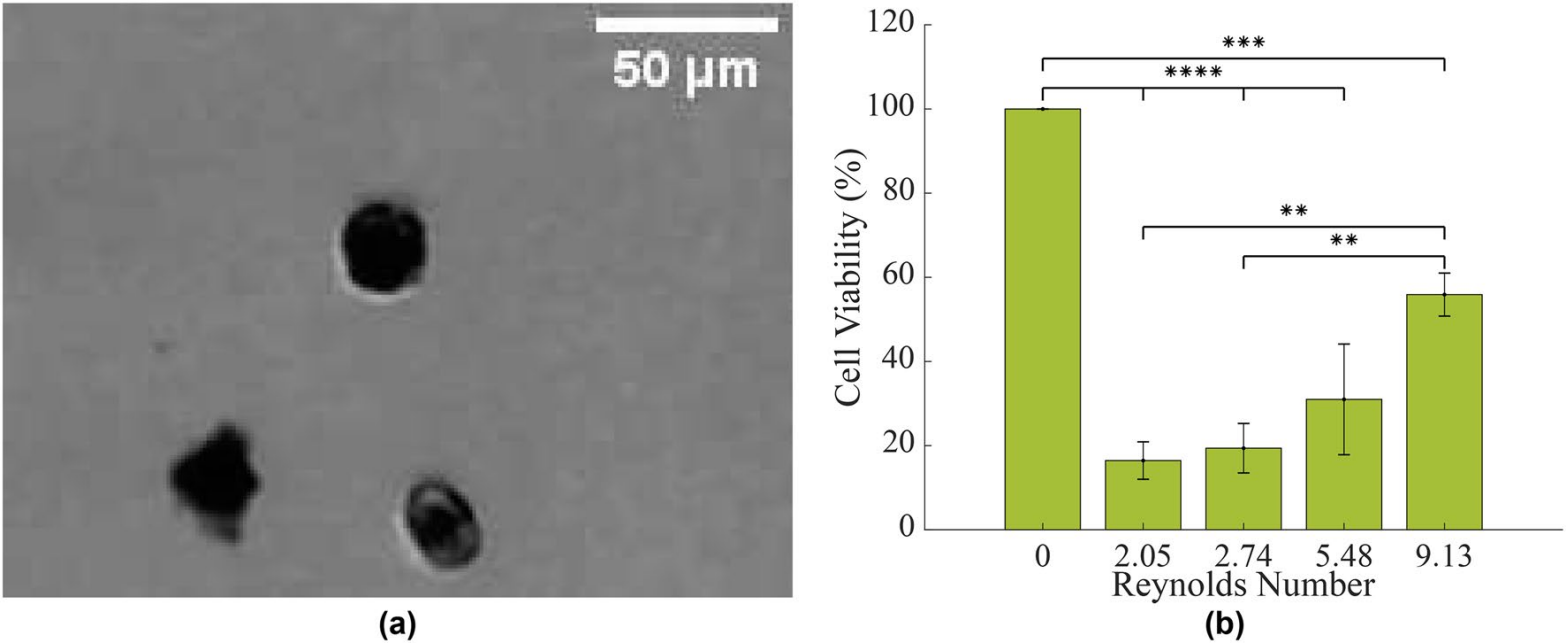
**(a)** Primary fibroblasts following suspension in Poiseuille flows of $Re=$ 5.48 for 24 hours, stained with trypan blue. **(b)** The viability of primary intestinal fibroblasts in circulation, assessed using MTT assay. Statistical analysis was conducted using two-way ANOVA. Results were normalised to static cells in suspension (or *Re* of 0). ****$p<$ 0.0001, ***$p<$ 0.001, **$p<$ 0.01, $n=$ 3 replicates, vertical bars represent the s.e.m.

Again, cell viability was assessed using MTT, as outlined previously. An image of the fibroblasts following exposure to $Re=$ 5.48 and stained by trypan blue is shown in Fig. 3a and the results are shown in the bar graph in Fig. 3b.

The MTT results showed a significant difference between the viability of the control cells and those of all other test conditions ($p=$ 0.0009 in comparison with $Re=$ 9.13 and $p<$ 0.0001 in comparison with all other *Re*). Like the T-cells previously, the viability of intestinal fibroblasts also increased with an increasing flow rate, however viabilities were the lowest of all the tested cell types. As this trend was observed in more than one cell line, it is explored in further detail later. Intestinal fibroblasts suspended in fluid flow of 11.3 $\upmu$L/min recorded viabilities of 16 ± 4%, while at higher flow rates of 50.3 $\upmu$L/min, cell viability remained at 56 ± 5%. As the $\tau _w$ that intestinal fibroblasts are normally exposed to is an order of magnitude below what was investigated in this study, it may explain the high death rate, however, they also indicate that there are active survival mechanisms in professional advecting cells to allow them to maintain viability in flow.

These findings have implications for fibroblast research. Many cell isolation protocols utilize flow cytometry^38^ while FACS are used for analysis purposes. These methods may need to be reviewed as they may be having detrimental effects on the cells. In light of the above findings, it may be more appropriate to process them at higher flow rates which appear more conducive to cell viability.

### Viability of SH-SY5Y neuroblastoma cells in Poiseuille flow

**Figure 4:**
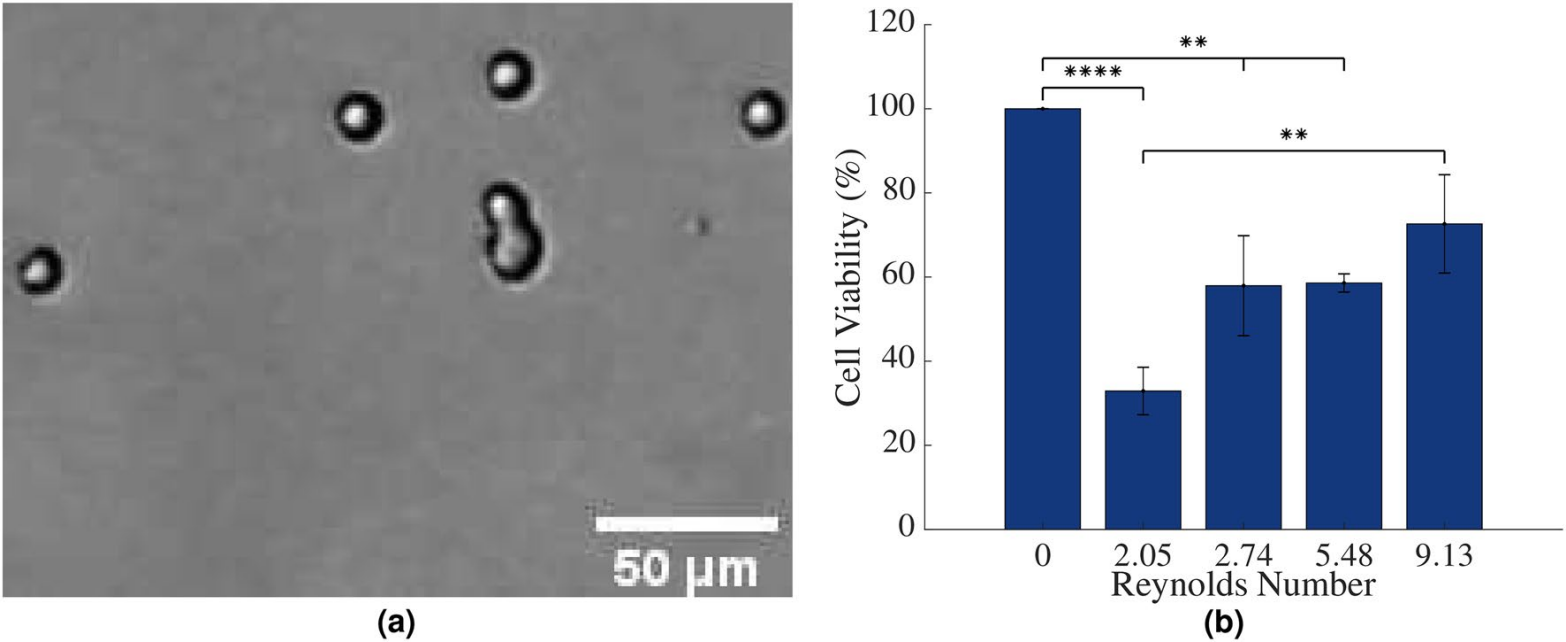
**(a)** SH-SY5Y neuroblastoma cells following suspension in Poiseuille flows of $Re=$ 2.74 for 24 h, stained with trypan blue. **(b)** The viability of SH-SY5Y neuroblastoma cells in circulation, assessed using MTT assay. Statistical analysis was conducted using two-way ANOVA. Results were normalised to static cells in suspension (or *Re* of 0). ****$p<$ 0.0001, **$p<$ 0.01, $n=$ 3 replicates, vertical bars represent the s.e.m.

Finally, it was decided to evaluate whether the survival characteristics of cancer cells observed so far were broadly applicable across all cancer types or whether they were similarity partitioned, like primary cells, into those that normally experience flow and those for which suspension in flow is detrimental to their viability. To do this, the viability of SH-SY5Y neuroblastoma cells exposed to Poiseuille flow for 24 h was investigated. Interestingly, studies which have used the expression of keratin *KRT19* as a measure of the metastatic potential of different cancer cell lines have found that it is overexpressed in breast cancer cells, such as MCF-7 and MDA-MB-231 cells in comparison with other cancer cell types and is very low specifically in SH-SY5Y cells^39^, indicating lower metastatic capabilities in the neuroblastoma cell line, and therefore, more of an affinity to static cellular microenvironments like the primary fibroblasts (see ‘Tumour Cells’ in Fig. 1). Again, cell viability was assessed using MTT as outlined previously. An image of the neuroblastoma cells following exposure to $Re=$ 2.74 and stained by trypan blue is shown in Fig. 4a and the results are shown in the bar graph in Fig. 4b.

The MTT results showed that the viability of cells under *Re* of 2.05, 2.74 and 5.48 were significantly different to the control viability ($p<$ 0.0001, $p=$ 0.0042 and $p=$ 0.0048 respectively). Like the previous cells, the viability of the neuroblastoma cells again appeared to increase slightly with an increasing flow rate and *Re*. In an in vivo environment, neuroblastoma cells are typically only exposed to interstitial flow which results in $\tau _w$ values of $\approx$ 0.4 Pa^36^, much lower than those investigated in this study. At the lowest flow rates, closest to interstitial flow, at $Re=$ 2.05, inducing a $\tau _w$ value of 2.25 Pa, the viability of the SH-SY5Y cells was 33 ± 6%, while at higher flow rates, of $Re=$ 9.13, cell viability remained at 73 ± 12%. While again, the viability of the neuroblastoma cells increased very slightly across the different laminar flow conditions, the overall viability of these cells following their exposure to flow conditions were lower than the two previously tested breast cancer cell lines as well as the T-cells.

### Comparative analysis of different cell types

**Figure 5:**
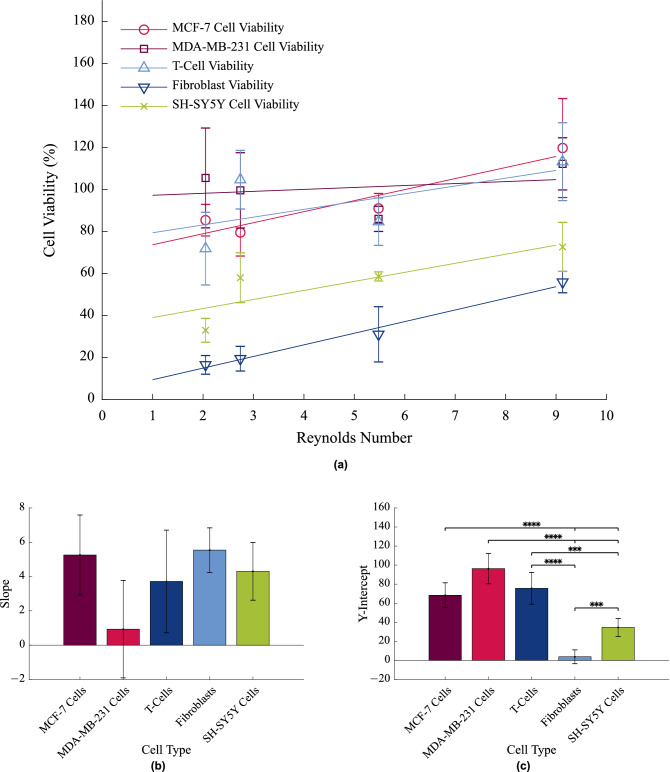
**(a)** The viabilities of MCF-7 and MDA-MB-231 breast cancer cells^10^, primary T-cells, primary porcine intestinal fibroblasts and SH-SY5Y neuroblastoma cells in circulation with fitted viability lines. All viabilities are presented as a percentage of the control viability. **(b)** A comparative analysis of the slopes of viability. Statistical analysis was conducted using linear regression analysis. Vertical bars represent the s.e.m. **(c)** A comparative analysis of the elevations of viability. Statistical analysis was conducted using linear regression analysis. ****$p<$ 0.0001, ***$p<$ 0.001, vertical bars represent the s.e.m.

As seen previously throughout the different cell studies, differences were observed between the viability of different cell types. Furthermore, the authors wished to establish if a relationship existed between an increasing *Re* and cell viability, and if so, could this trend be used to predict cell viability at *Re* outside of the scope of this study. This information could then be used to inform future experimental parameters or the boundary conditions within which cell viability is unaffected. For this reason, a comparative analysis of the viability of the T-cells, intestinal fibroblasts and neuroblastoma cells was conducted using linear regression and compared to those of breast cancer cells, investigated previously^10^. These are shown in Fig. 5a. Comparative analysis of the line slopes and elevations are also shown in Fig. 5b,c respectively.

While the viabilities of the two breast cancer cell lines and the primary T-cells remained high throughout the experiment ($\approx$ 80–100% viability in comparison to the control), the viability of the neuroblastoma cell line, cells that are not typically suspended in *Re* as high as the aforementioned cells, was significantly lower, typically at 40–60% (elevation of $p<$ 0.0001 in comparison with both MCF-7 and MDA-MB-231 cells, and $p=$ 0.0005 in comparison with T-cells, as seen in Fig. 5c). Furthermore, the viability of the primary fibroblasts, of approximately 15–40% was significantly lower than all other cell types (elevation of $p<$ 0.0001 in comparison with MCF-7, MDA-MB-231 cells and T-cells, and $p=$ 0.0003 in comparison with SH-SY5Y cells, as seen in Fig. 5c). Cell survival cues in tissue are continually re-enforced by contact with the surrounding microenvironment. A reduction in this pro-survival signal may therefore contribute to the sensitivity observed in fibroblasts in this study. It is therefore interesting to note that the breast cancer cell lines were able to quickly adapt to their altered conditions to survive at very high levels suspended in flow. It is likely that alternative survival mechanisms have been acquired by metastatic cells to allow them to transit the cardiovascular and lymphatic systems, indicating that these cells have the ability to overcome the sensitivity that characterised the fibroblast response. This could identify these survival mechanisms as relevant targets of therapeutic potential.

**Figure 6:**
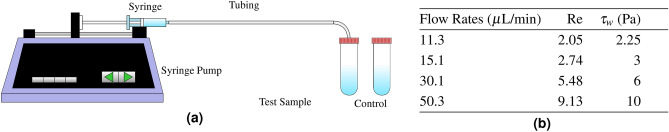
**(a)** The experimental apparatus, consisting of a syringe pump infusing cells through the tubing. Figure was created using Inkscape^31^. **(b)** Investigated flow rates and corresponding $\tau _w$ and Reynolds numbers (Re) used in circulatory experiments.

In MDA-MB-231 cells and primary T-cells, a linear regression analysis showed that viability was unaffected by an increasing *Re* (they were found to be not significantly non-zero), however a very small increase in viability was statistically significant in the MCF-7 cells, the primary fibroblasts, and the SH-SY5Y cells ($p=$ 0.0471, $p=$ 0.0016, and $p=$ 0.0284 respectively). This was somewhat surprising as, according to the wall shear stress equation, an increase in the flow rate will lead to an increase in $\tau _w$. Additionally, as seen previously, an increase in laminar shear stress on static cells results in decreased cell viability^10^. Interestingly, this slight increase was reflected across all cell types with no significant differences observed between the line slopes of different cell types, as seen in Fig. 5b. A similar slight increase in the viability of suspended colon cancer cells with increasing $\tau _w$ over a constant time frame in a continuous flow circuit has also been previously observed^15^. In this study, colon cancer cells were circulated through a microfluidic device with a 800 $\upmu$m long constriction by a peristaltic pump for 20 h. The constriction resulted in $\tau _w$ values of 0–6.05 Pa, which are comparable to those used in this study (0–10 Pa, see Fig. 6b), and *Re* of up to 0.372, which are an order of magnitude less than those presented in this investigation.

In their study, Fan et al. observed that cell viability decreased over its 20 h duration, to values less than those observed in this study, however this could be due to the peristaltic pump, rather than the fluidic conditions that the cells were subjected to^10^. What is particularly interesting, however, is that within each time point after 1 h, there was an increase in cell viability with an increase in flow rate, findings that are consistent with the results presented in this investigation, despite a different pump-delivery mechanism. The authors of this study hypothesized that this was due to different regulation mechanisms of intracellular signalling of cells in suspension^15^ and later studies have echoed this further, finding that circulatory shear stress induces molecular changes, altering gene expressions which are key regulators in the epithelial-to-mesenchymal transition process^40,41^. Furthermore, another study carried out by Suwannaphan et al. observed a similar increase in leukocyte viability with an increasing flow rate in spiral microchannels, however this may also be attributed to an increased time duration in the channel at lower flow rates^7^.

The positive slope observed in these studies, as well as that observed in all cells in this study, point to the development of a protective effect within the microchannel with an increase in *Re*. The elevations of the viability were different, depending on the cell type, pointing to a type-dependant sensitivity to *Re*. A similar cell type-dependant response has previously been observed by studies examining the extent of cell damage following circulation in a microfluidic device^42^. However, there was no significant differences observed between the slopes of the viability between cell types and all slopes were were positive, indicating a correlation between increasing *Re* and increasing viability. This points to a broadly applicable effect of increasing viability due to increasing *Re*. As observed previously, this could be due to an increased focusing effect of cells at the channel centre at higher *Re*^10^, shielding them from potentially damaging force effects closer to the channel wall. This implies that an increase in the *Re* will have a universal benefit for all cell types suspended in fluid flow in microfluidic devices as it correlates with an increasing viability. Cell specific regimes therefore need to be designed to align with optimal cell viability in accordance with these findings, that is, they should be operated at high *Re* for maximal benefit. The limits of this positive correlation, however, have not been determined in this study but may be of interest in future work, particularly given the positional locations.

In this study, it was hypothesized that cells migrated to inertial positions that shielded them from high levels of $\tau _w$, allowing them to survive very high flow rates. However, what is also possible are that the intrinsic differences within each cell type are responsible for the widely differing viabilities between cell types. These mechanisms allow cells to survive in their native environments or, in the case of breast cancer cells, allow them to easily adapt to a changing environment to allow them to thrive in different locations. It also furthers the argument that cell viability is not influenced at all by $\tau _w$. Further study is therefore required in order to investigate if other factors can be used to more accurately predict cell viability in microchannels such as $\nabla \tau _p$ or the shear gradient across the channel width, as has been previously suggested^10,11^. Previous studies have also shown that the location occupied by suspended cells in a channel can be dictated by the deformation properties of that cell, a property which is unique to each cell line^43^. It remains to be seen whether this location influences $\nabla \tau _p$ or the shear gradient acting on the cell and therefore its viability. It may also be of interest to examine the cell signalling mechanisms employed by the cells possessing a high survival rate, particularly the breast cancer cells which were able to adapt to a suspension environment. In such a study, it would be of use to compare these mechanisms in both cells that possessed the ability to survive in comparison to those that did not. This may provide insight into how other traditionally static cells, such as fibroblasts, may be adapted to survive similar microfluidic assays which might, counter-intuitively, be achieved through increasing the flow rate through such devices. Furthermore, this study indicates that flow adaptation could be a hallmark of metastatic disease, allowing solid tissue derived cells to spread, which could provide a potential therapeutic target of cell disruption.

When interpreting the results of this study, certain limitations should be taken into consideration. While these results provide a good benchmark for cell viability in straight, in vitro microchannels under laminar flow conditions, similar to current standard flow cytometry and adoptive cell therapy procedures, it is still a fundamental study in the context of the body’s circulatory systems which do not conform to these parameters, and so, does not necessarily represent the reactions of different cell types to in vivo fluid dynamics. Future studies in excised vessels are required to expand further on this study. Additionally, some flow systems may use much higher concentrations of cells, which can cause effects due to cell-cell collisions to become prominent. This would also be useful to investigate in future studies, as it is highly possible that this will have a negative effect on cell viability. Furthermore, it not known if the sensing and survival pathways or indeed the cell death pathways are common between healthy and cancerous cells. Future work may focus on pathway analysis, as well as investigating apoptosis or necrosis mechanisms which have been activated. Finally, while the results of this study show an increased viability with an increasing flow rate, it may be that above a certain threshold, the cells are fully focused and no longer benefit from an increasing *Re*. Indeed, beyond this, it is possible that cells may experience a reduction in viability again as the flow rate imparts greater forces on the cells. Further studies are required in order to ensure that this is not the case.

The results of this study have implications primarily in in vitro cell techniques such as lab-on-a-chip devices, flow cytometry and CAR T-cell therapy. From these results, it can be concluded that the former two techniques, primarily used for both research and diagnostic purposes, may produce erroneous results due to cell damage from the fluidic forces in the microchannels, particularly in the analysis of cells whose native environment is not that of a circulating cell or in suspension. It is therefore important to ensure that fluidic conditions in such techniques are not contributing to cell death before analysis. This outcome is also particularly important in the case of the cell therapies, where cells are required to be returned to the body and so cannot be defective in any way, potentially causing further harm to a patient with an already compromised immune system or depleting stocks of an already limited source of T-cells. As it is unlikely that cells will be exposed to these conditions for periods longer than 24 h, researchers and health care professionals can be confidant that these techniques are not causing a decrease in T-cell viability. Finally, such results may point to the potential target of flow adaptation for cancer therapeutics.

## Conclusion

An experimental study was carried out to investigate the viability of different cell lines and primary cells in suspension under different Poiseuille flow conditions in a microchannel. It was found that after a circulation period of 24 h there was no drop in the viability of T-cells. The viability of neuroblastoma cells and primary intestinal fibroblasts were significantly lower than other cell types. Additionally, there was an increase in cell viability with increasing flow rate in three of the five cell types.

These results reflect similar findings in breast cancer cells, confirming that wall shear stress is not a good indicator for the viability of circulating cells and that the local shear distribution on the cell surface, or across the channel width, may be a better gauge, however, further investigations are required in order to determine if this is the case.

The results indicate that different cells have different levels of sensitivity, implying that in cell isolation regimes and separation methods, parameters will need to tailored to the individual cell population under investigation, most likely by increasing the flow rates used in such environments. It also shows that there exists both cell types that can be considered to be ‘professional advectors’ who are insensitive to different flow rates while others, more used to a static environment, are significantly impacted upon being suspended in flow. These results have implications for lab-on-a-chip devices, flow cytometry and, in particular, CAR T-cell therapy, where the viability of cells following suspension in laminar flow in microchannels is critical. Furthermore, they have implications in the targeted treatment of tumour cells which have the ability to readily adapt to flow conditions.

## Methods

### T-cell acquisition

Whole blood was acquired from three healthy adult volunteers and added to lithium heparin (Vacutest Blood Collection Tubes, VWR, Dublin, Ireland) in order to prevent coagulation. T-cells were then separated from the blood using EasySep Direct Human T Cell Isolation Kit (STEMCELL, Cambridge, UK), along with the EasySep Magnet (STEMCELL, Cambridge, UK). T-cell numbers and sizes were measured prior to experiments using a LUNA Automated Cell Counter (Logos Biosystems Inc., Villeneuve d’Ascq, France), and were found to have an average diameter of 8.89 ± 0.61 $\upmu$m.

### Cell lines and cell culture

Two cell lines were used; SH-SY5Y neuroblastoma cells (Sigma-Aldrich Inc., Arklow, Ireland) and neonatal porcine primary small intestinal fibroblasts (PELOBiotech, Munich, Germany) in order to generate data from a wide variety of different cell types. SH-SY5Y cells were maintained in cell media consisting of 50% Dulbecco’s modified Eagles medium (DMEM), (Sigma-Aldrich Inc., Arklow, Ireland), and 50% Ham’s nutrient mixture F12 (Sigma-Aldrich Inc., Arklow, Ireland), supplemented with 10% foetal bovine serum (FBS), (Sigma-Aldrich Inc., Arklow, Ireland) and 1% penicillin/streptomycin (Sigma-Aldrich Inc., Arklow, Ireland). Intestinal fibroblasts were maintained in fibroblast basal medium (PELOBiotech, Munich, Germany), supplemented with 10% FBS (PELOBiotech, Munich, Germany), 1% antibiotic-antimycotic solution (PELOBiotech, Munich, Germany), 1% L-glutamine (PELOBiotech, Munich, Germany), 0.1% hydrocortisone (PELOBiotech, Munich, Germany) and 0.1% fibroblast growth factor (FGF), (PELOBiotech, Munich, Germany). Both cell lines were cultured in an incubator at 37 $^{\circ }$C and 5% CO$_{2}$. Cell numbers and sizes were measured prior to experiments using a LUNA Automated Cell Counter. SH-SY5Y cells were found to have an average diameter of 15.8 ± 0.6 $\upmu$m while intestinal fibroblasts were measured to be 13.7 ± 0.3$\upmu$m in diameter.

### Cell preparation

Before experimentation, all cell types were counted using a LUNA Automated Cell Counter and resuspended at a concentration of approximately 750 cells/$\upmu$L in serum-free Dulbecco’s Modified Eagles Medium (DMEM, Sigma-Aldrich Inc, Dublin, Ireland) and 20% Percoll (Sigma-Aldrich Inc, Dublin, Ireland). This has previously been found to be the optimal percentage of Percoll to prevent cell settling and adhesion^43^ and has also previously been shown to have no effect on cell viability^44^. The lower limit of the cell concentration used was dictated by the number of cells required to produce an accurate result following sample acquisition from the cell suspension reservoir, while the upper limit was dictated by the need to reduce the effects due to cell-cell interactions. Previous studies have advised the use of volume fractions of < 1% in order to minimise these effects^45–47^. Indeed, those examining hematocrit levels of RBCs found that activity due to this phenomenon was minimal below a volume fraction of 0.5–2%^4,48–51^. Therefore, our experimental setup utilised a volume fraction of cells of 0.32%. Mixed solutions of cell media and Percoll with very low concentrations of cells have previously been found to act as a Newtonian fluid^52^.

### Experimental apparatus

A cell suspension of approximately 1 mL was placed in a syringe while a further 1 mL was placed in a control vial. A syringe pump (Pump 11 Elite, Harvard Apparatus, Cambourne, UK) was then used to infuse the cell suspension through the in vitro model at a constant flow rate. When the cell suspension was completely injected from the syringe, the pump was programmed to withdraw the solution again. This infusion and withdrawal process was repeated over a 24 h time period. A similar method has previously been used to examine the extent of cell damage on suspended cell populations^42,53^. Figure 6a displays the experimental set-up used in the circulatory experiments. The microchannel consisted of 100 $\upmu$m inner diameter, perfluoroalkoxy alkane (PFA) tubing (Cluzeau Info Labo, Sainte-Foy-la-Grande, France) and was $\approx$30 cm in length. Different flow rates were used to infuse the cells through the channel in order to expose them to different levels of hydrodynamic forces (See Fig. 6b). The entire set-up was placed in an incubator at 37 $^{\circ }$C and 5% CO$_2$. The control vial was also placed in the incubator for this period of time. Immediately following the experiment, both the control and test samples were assessed for viability.

### Viability acquisition and statistical analysis

The viability of different cells was assessed using an MTT assay as it was believed to be one of the most accurate methods^54^. Cell samples were placed in a 96-well plate (Fisher Scientific Ireland Ltd., Dublin, Ireland) in serum-free media. MTT solution, composed of 5 mg/mL of 3-(4,5-dimethylthiazol-2-yl)-2,5-diphenyltetrazolium bromide (MTT) (Fisher Scientific Ireland Ltd., Dublin, Ireland) in PBS, was added to the cells which were then incubated for 2 hours at 37 $^{\circ }$C and 5% CO$_2$. In this time, the MTT is reduced by viable cells to purple formazan. This is then solubilised by adding a solubilisation buffer to the cell solution. The solubilisation buffer consists of 0.1 gm/mL of sodium dodecyl sulphate (SDS) (Sigma-Aldrich Inc., Arklow, Ireland) in 0.01M of Hydrochloric Acid (Sigma-Aldrich Inc., Arklow, Ireland). Cells were incubated again for 4 h before the absorbance was measured at 570 nm on a spectrophotometer (Synergy H1, BioTek Instruments Inc., Swindon, UK). As absorbance is proportional to cell viability, the amount of viable cells was calculated. For each cell line, each experiment was repeated at least three times. For T-cells, each experiment was repeated at least three times for each donor (or nine times in total) and all data is presented as ± standard errors from the mean. Statistical analysis within cell types was conducted using ANOVA, and two sample unequal variances were used to calculate the p-values between groups. Statistical analysis between cell types was conducted using linear regression. All cell viability percentages are presented as percentages of the control viability.

### Ethics approval and consent to participate

The study protocol was approved by the ethics committee in the University of Limerick and all experiments were conducted in accordance with the guidance and regulations as specified by the University of Limerick. Informed consent was obtained from all study participants. The study was performed in accordance with the Declaration of Helsinki.

## Data Availability

The datasets used and/or analysed during the current study are available from the corresponding author on reasonable request.
